# Mesenchymal Stem Cells as a Potent Cell Source for Bone Regeneration

**DOI:** 10.1155/2012/980353

**Published:** 2012-02-16

**Authors:** Elham Zomorodian, Mohamadreza Baghaban Eslaminejad

**Affiliations:** Department of Stem Cell and Developmental Biology, Royan Institute for Stem Cell Biology and Technology, ACECR, 1665659911 Tehran, Iran

## Abstract

While small bone defects heal spontaneously, large bone defects need surgical intervention for bone transplantation. Autologous bone grafts are the best and safest strategy for bone repair. An alternative method is to use allogenic bone graft. Both methods have limitations, particularly when bone defects are of a critical size. In these cases, bone constructs created by tissue engineering technologies are of utmost importance. Cells are one main component in the manufacture of bone construct. A few cell types, including embryonic stem cells (ESCs), adult osteoblast, and adult stem cells, can be used for this purpose. Mesenchymal stem cells (MSCs), as adult stem cells, possess characteristics that make them good candidate for bone repair. This paper discusses different aspects of MSCs that render them an appropriate cell type for clinical use to promote bone regeneration.

## 1. Introduction

Bone is a highly specific, dynamic tissue capable of maintaining viability under mechanical stress and external continuous compression. This capability of bone tissue diminishes with increasing age [1]. Furthermore, small bone damage can repair spontaneously without intervention. However, if there is extensive bone damage due to pathologic and traumatic injuries, there will be a need for reconstructive surgery and bone transplantation. In this regard, autologous tissue transplantation would be the best and safest strategy for bone repair. Autologous bone graft is taken from the patient's own iliac crest, ribs, or calvarium. Unfortunately, access to autologous bone graft is limited. Furthermore, obtaining an autograft is associated with morbidity, pain, and infection at the donor site. Because of such disadvantages other alternatives are needed [2, 3]. Allogenic bone tissue implantation may be chosen to repair large bone defects, but this bone substitute also exhibits several drawbacks, which include the possibility of disease transmission, graft rejection, problems with graft integration and viability at the recipient site [4]. Emergence of modern bone engineering strategies based on osteogenic cells, osteoinductive stimulator, and osteoconductive scaffolds are recognized as potential ways to create biologic tissue substitutes for regenerating large bone defects [5]. The choice of cell sources that can efficiently differentiate into bone tissue is the first, important step during bone engineering. Several cell types can potentially be used as cellular components in bone engineering. These include osteoblast, embryonic, and adult stem cells. Among these candidates, mesenchymal stem cells (MSCs) as adult stem cells possess some characteristics that make them more appropriate for use in promoting bone regeneration.

Historically, the definitive presence of MSCs was discovered about 40 years ago by Friedenstein et al. in bone marrow tissue. They described these cells as mononuclear nonphagocytic cells with fibroblast-like phenotype and colongenic potential capable of adhering to the culture surface in a monolayer culture [6]. Later, it has been shown that MSC-like population were present in a wide range of adult tissues, including trabecular bone [7], synovium [8], adipose tissue [9], skeletal muscle [10], periosteum [11], dermis [12], blood [13, 14], deciduous teeth [15], amniotic fluid [16], and umbilical cord blood [17]. Currently, good manufacturing practice (GMP) has been developed to produce the cells for use in clinic [18].

It should be mentioned that stem cells are defined by two key characteristics: the ability of multilineage differentiation and the capacity of self-renewal [19]. Of these MSCs possess multilineage differentiation potential but have a limited proliferation capacity since they enter senescence after a few population doubling in culture [20, 21]. Therefore they cannot be considered true stem cells. For this reason, in related literatures the cells have been referred to as by different terminology as colony-forming unit fibroblasts (CFU-Fs), mesenchymal stromal cells (MSCs), marrow stromal cells (MSCs), marrow progenitor cells (MPCs), and marrow stromal fibroblasts (MSFs) [22–28]. Nowadays, the term mesenchymal stem cells is the dominant term most frequently used by investigators. Here, the specific characteristics that make MSCs promising cells for use in bone regeneration strategies will be discussed.

## 2. MSCs Escape Ethical Concerns

Among candidate cells for bone regeneration, embryonic stem cells (ESCs) possess ethical issues limiting their application in bone regeneration. ESCs are derived from the blastocyst inner cell mass and can be directed toward differentiation into varying cell lineages, including osteoblastic cell lineages under suitable culture conditions [29–32]. To date, multiple studies have been conducted on ESCs osteogenic differentiation in vitro and their application in bone tissue engineering with varying scaffolds. For example, it has been shown that culturing ESCs on poly-lactide-co-glycolic (PLGA) or nanofibers made from PLLA (poly (l-lactic acid)) is associated with high expressions of osteogenic markers, including alkaline phosphatase and osteocalcin [33, 34]. Despite increasing interest in the application of ESCs in bone engineering technology, research is highly limited due to political issues as well as ethical concerns associated with these cells. The primary concern is the source from which these cells are derived. The use of excess embryos produced in IVF to create ESCs is not acceptable according to religious and ethical points of view. Additionally, some reports have indicated that transplantation of ESCs has led to teratoma formation in the animal model [35, 36]. For these reasons postnatal adult stem cells, including MSCs that could be derived from a patient's own tissues and do not possess ethical limitations, are considered more appropriate for clinical use.

## 3. MSCs Are Residents of Multiple Tissues

MSCs have been reported to constitute about 0.01%–0.001% of the marrow mononuclear population [37]. These cells can be isolated from marrow aspirates of the superior iliac crest, femur, and tibia. For this purpose, marrow cells are usually enriched for mononuclear cells with Ficoll or Percol and then plated on culture plastic vessels in order to prepare adherent cell populations [38]. It has recently been demonstrated that late plastic adherent MSCs possess higher osteogenic potential [39]. Alternatively, MSCs can be obtained by the preparation of a population positive for STRO-1 or CD105. It has been reported that a population negative for CD45 or Gly-A are from MSCs [40]. By now, many researchers have studied optimized culture and differentiation of MSCs in vitro and their application in regenerating bone defects in animal models and humans [41–44]. Since collection of bone marrow is invasive and expansion and osteogenic differentiation of marrow-derived MSC seem to be reduced with advancing age, investigators have attempted to find other tissue sources for MSCs [45]. According to research, multiple tissues have been found to contain MSC-like population; of these, adipose tissue as well as birth-associated tissues, including umbilical cord and dental pulp, has gained considerable attention.

The presence of cells with multipotent differentiation capacity in adipose tissue is promising due to the ease of accessibility of adipose tissue and its abundance in the body. Adipose tissue can be an appropriate substitute for marrow in regenerative medicine and tissue engineering [46, 47]. Adipose-derived stromal cells (ADSCs) can be derived from adipose collected by liposuction and lipectomy [48]. ADSCs are able to maintain proliferation potential as well as differentiation capacity even in older people. The differentiation potential of ADSCs is largely dependent on the concentration of ascorbic acid and dexamethasone in culture medium [49, 50]. By now, many studies conducted on animal models have confirmed the regenerative potential of ADSCs in bone defects. The first report regarding repair and production of bone tissue in vivo belongs to Lee et al. who transplanted ADSCs loaded onto PLGA [51]. Later, Hicok et al. have noted the production of osteoid matrix when a combination of ADSCs, hydroxyapatite (HA) and tricalcium phosphate (TCP), were transplanted in nude mice [52]. In 2004, these cells were used for the repair of human calvarial defects [53]. To date the effect of various biomaterials, including HA, human cancellous bone fragments, deproteinized bovine bone granules, and titanium, has been investigated in terms of ADSC attachment, proliferation, and differentiation [54, 55].

The umbilical cord from a newborn baby contains two arteries and a vein covered with mucus connective tissue rich in hyaluronic acid, referred to as Wharton's jelly. According to studies, MSC-like cells can be derived from various components of this cord [56]. For example, blood from an umbilical cord is a rich source for pluripotent cells which are also referred to as umbilical-cord-blood-derived MSCs (UCB-MSCs). These cells are quite similar to marrow-derived MSCs and have osteogenic potential in an optimized culture [57–59]. Many investigations have thus far been conducted on bone engineering by using these cells and various scaffolds [60, 61].

Several stem cell types in dental tissue have been reported including dental pulp stem cells (DPSCs), stem cells from human exfoliated deciduous teeth (SHED), stem cells of the apical papilla (SCAP), periodontal ligament stem cells (PDLSCs), and dental follicle progenitor cells (DFPCs) [15, 62]. Since DPSCs can be easily isolated by enzymatic digestion of pulp tissue many studies have been conducted regarding bone engineering with these cells and appropriate 3D scaffolds, including HA/TCP and polylactic-co-glycolic acid (PLGA) [63, 64].

## 4. MSCs Can Efficiently Differentiate along an Osteogenic Lineage

MSCs osteogenic property was the first reported differentiation capacity when they were discovered. Indeed, even prior to definitive isolation of MSCs from bone marrow, some transplantation experiments clearly showed the osteogenic capacity of marrow tissue. Friedenstein et al. were the first to isolate and describe the cellular equivalent of osteogenic features of marrow tissue [65].

Osteogenic differentiation is a highly programmed process that consists of many stages including proliferation, differentiation, matrix deposition, mineralization, and matrix maturation. The general protocol for in vitro bone differentiation of MSCs involves incubation of cell monolayer in a culture medium containing dexamethasone, beta glycerol phosphate, and ascorbic acid for a period of two to three weeks [66]. Dexamethasone is a synthetic glucocorticoid that stimulates MSC proliferation and is essential for their osteogenic differentiation [67, 68]. Although the mechanism of dexamethasone's effect is not well known, it has been speculated that this reagent exerts its effects through upregulation of the beta catenin-like molecule TAZ, which results in up-regulation of Runx2-related transcription factor and osteogenic differentiation [69]. The optimal concentration of this reagent for MSC bone differentiation is about 10 nM, which corresponds with its physiologic concentrations [70]. Organic phosphate released after enzymatic hydrolysis of beta glycerol phosphate plays an important role in matrix mineralization. This free phosphate is usually applied in 5–10 mM concentrations for MSC bone differentiation [71]. Ascorbic acid is a cofactor in the hydroxylation of prolins and lysine moiety of collagen molecules and is the abundant protein in ECM. This reagent is used in 50–500 *μ*M concentrations [72]. In addition to these osteogenic supplements, there are other osteogenic factors including (1,25-D3) 1,25-dihydroxyvitamin and BMPs (BMP2) [73].

MSC in vitro bone differentiation results from the activation of some well-known molecular signaling pathways. Each osteogenic reagent activates a molecular pathway that leads to a differentiated phenotype. Although the osteogenic effects of a number of these reagents have long been known, specific pathways by which the effects are mediated remain to be clarified. The activation of wingless-type MMTV integration site family of the protein (Wnt) signaling pathway [74], mitogen-activated protein kinase (MAPK) signaling pathway [75], TGF beta and BMP signaling pathways [76], and RHO-GTPase signaling pathway [77] has been established in MSC bone differentiation.

 Activation of signaling pathways by osteogenic supplements eventually leads to activation of osteoblast-specific signal proteins and specific osteoblastic transcription factors. Cbfa1 (core binding factor alpha 1) also called as Runx2 (runt-related gene 2) is one of the most studied transcription factors expressed in MSCs upon their commitment toward osteogenic differentiation [78, 79]. Runx2, as a master switch, adheres to osteoblast-specific cis-acting element (OSE2) in the gene promoter region stimulating the expression of bone-specific genes such as coll I, osteocalcin, osteopontin, and alkaline phosphatase [80]. Osterix is another transcription factor involved in MSC bone differentiation, which has been clearly shown in murine MSCs where they were retrovirally transduced with the osterix gene [81]. Addition of dexamethasone to the culture of murine calvarial osteoblasts has been reported to induce expression of osterix as well as Runx2 genes [82].

Optimal conditions for MSC in vitro bone differentiation are well been established. For example, the addition of rhBMP-2 to osteogenic medium can facilitate proliferation and osteogenic differentiation of BMSCs both in vitro and in vivo. The use of alpha MEM versus DMEM and application of low-passaged versus high-passaged cells can end with higher expression of osteogenic genes and more culture mineralization [83–85]. Studies on scaffold designing and the effect of biomaterial on bone repair have indicated that calcium phosphate-based scaffolds, including hydroxyapatite (HA) and tricalcium phosphate (TCP), are more appropriate for bone engineering due to their osteoconductive properties [86]. Applying fluid shear stress (FSS) on the MSC osteogenic culture increases the expression of bone-specific genes and deposition of mineralized matrix. FSS mediates its effects through regulation of mechanosensitive signaling molecules, including ion channel and integrins, which are able to convert mechanical into chemical signals [87, 88].

## 5. Nonimmunogenic Properties of MSCs

MSCs possess immunologically specific characteristics; therefore they would be general donors for therapeutic applications. Immunologic phenotypes of MSCs are MHC I+, MHC II−, CD 40−, CD80−, and CD 86− [89]. Graft rejection by the immune system occurs when T cells are fully activated. T cells require two signals to become fully activated. The first signal is provided through the T-cell receptor which interacts with peptide-MHC molecule 1 on the membrane of antigen-presenting cells (APC). A second signal, the costimulatory signal, is provided by the interaction between co-stimulatory molecules, including CD80 and CD86 that are expressed on the membrane of APC and the T cell [90]. MSCs do not trigger T-cell activation owing to the absence of CD80 and CD86 in their membrane [91]. The immunosuppressive nature of MSCs has been shown in skin allografts of baboon models.

According to research, MSCs secrete soluble factors that inhibit CD4+ and CD8+ T-cell activation as well as proliferation. Among these factors are indoleamine 2,3-dioxygenase (IDO), nitric oxide, TGF-beta, and prostaglandin E 2 [92–95]. It has been demonstrated that MSCs stop the B-cell cycle at the G0/G1 stage and inhibit their differentiation into plasma cells [96, 97]. Ramasamy et al. have indicated that BMSCs are able to inhibit dendritic cell (DC) differentiation and prevent them from entering into the cell cycle [98]. DCs are able to efficiently present antigens to lymphocytes. According to research, monocytes differentiate into DCs in the presence of MSCs [99].

Immunomodulatory features of MSCs make them an important cellular candidate for cell-based treatment of tissue defects in an allogenic setting. For this reason, there is hope that MSCs could replace autologous and allogenic bone grafts which have known exhibited limitations in terms of availability and risk of pathogen transmission, respectively. At the allogenic approach, it will be possible to develop a cell bank to maintain MSCs from every donor for use in cell therapy. Prior to routine application of the cells in the clinic, an exact understanding of the immunologic features of MSCs and the underlying mechanism of action is needed [100, 101].

## 6. Injury-Seeking Capability of MSCs

One of the most important capabilities of MSCs is their migration capacity in response to signals produced by an injured bone [102, 103]. At the injury site, MSCs could possibly help with repair in two ways: (1) they differentiate to tissue cells in order to restore lost morphology as well as function, and (2) MSCs secrete a wide spectrum of bioactive factors that help to create a repair environment by possessing antiapoptotic effects, immunoregulatory function, and the stimulation of endothelial progenitor cell proliferation [103].

The precise mechanisms of cell trafficking in blood, transmigration through endothelial cell, and homing to the injured site are not thoroughly understood, but it has been speculated that chemokines and their receptors regulate this process [89]. Chemokines (chemotactic cytokines) are small proteins (8–10 KDs) with a capacity for creating a chemical environment appropriate for the migration of lymphocytes, neutrophils, and other immune cells towards inflammation, angiogenesis, and the organogenesis site. On the other hand, MSCs express a series of chemokine receptors that play a role in their migration in response to a chemokine gradient produced at the damaged site. These chemokine receptors include CCR1, CCR7, CCR9, CCR3, CCR4, CCR5, and CX3CR1 [104]. CXCR4 receptor and its specific chemokine (stromal cell-derived factor 1 (SDF1)) play an important role in stem cell trafficking, particularly HSCs [105]. It has been proposed that SDF1/CXCR4 could be a homing signal for MSCs in bone repair.

Kitaori et al. have reported that SDF1 expressed by periosteum mediates bone repair in the murine femoral model by recruiting MSCs to the fracture site [106]. The SDF1 gradient causes both host as well as infused MSCs to migrate towards the injured area. MSC migration has been proven in clinical trials performed by Horwitz et al. in which MSCs were injected to regenerate bone in six patients who suffered from osteogenesis imperfecta, where osteoblasts secrete defective collagen I resulting in osteopenia. Observations indicated that in 5 out of 6 children who received allogenic MSCs, cell migration to various tissues that included bone, skin, and marrow stroma was observed [107]. Transplantation of MSCs was followed by increased formation of compact bone and reduction in fracture frequency.

Considering the relationships of cell migration with the chemokine concentration gradient, it can be concluded that the application of MSCs must be performed at the time when the chemokine concentration gradient is established at an adjacent area to the injured site.

## 7. MSCs as Vehicles for Bone Gene Therapy

MSCs could be ideal carriers for therapeutic genes at a cell-mediated gene delivery strategy owing to their unique characteristics that include ease of isolation, culture, and expansion as well as their immunomodulatory property [108].

In the normal process of bone development and repair, cytokines and osteoinductive growth factors play a major role by recruiting osteogenic progenitors at the bone formation site and promoting their differentiation into bone cell lineages [109]. Therefore, the application of such factors which include related recombinant growth factors in large areas of bone damage would enhance new bone formation. However the problem is that recombinant growth factors have a limited half-life that limits their sustained supply into damaged tissue. To overcome this limitation, gene transfer strategies using cellular carriers have been proposed. This strategy offers the sustained delivery of the osteogenic factor to the damaged area [110]. Genetic manipulation of MSCs can be achieved by transduction using viral vectors such as the adenovirus (Ad) [111] or transfection by nonviral vectors such as liposomes [112]. Viral vectors have the advantage of high efficiency but trigger the immune system. In addition, they possess varying capacity to transfer genes into dividing and nondividing cells [113]. Non-viral vectors possess the advantage of not being toxic [114].

Many investigators have tried to regenerate bone by transfecting MSCs with the BMP gene. For example, Lieberman et al. have indicated that autologous BMSCs expressing Ad-BMP2 can considerably promote segmental femoral defects in rat models when compared with BMSCs expressing Ad-LacZ [115]. Transplantation of Ad-BMP2-MSCs in rabbits has been reported to be associated with new bone formation [116]. In spite of the multiple studies that have focused on temporary expression of factors using the adenovirus vector, Gysin et al. have observed permanent expression of BMP4 using retrovirus in BMSCs which lead to repair of critical sized calvarial defects in rats [117]. In one study, Lin et al. have compared BMP4-transfected MSCs from marrow and adipose tissue in bone repair of a rabbit model and found no significant difference [118].

It has been shown that Ad-Runx2-MSCs transplanted in murine calvarial defects produce more bone tissue compared to MSCs [119]. Recent studies have focused on simultaneous application of BMPs and RUNX2. When these two factors were entered into an immortal MSCs line and injected into mice, considerable bony ossicle with marrow cavity was observed (compared to the application of cells that expressed Ad-BMP2) [120]. Although no clinical trial to date has been conducted using genetically modified MSCs, studies have indicated that such a strategy would be more effective in enhancing bone repair.

## 8. Conclusion

MSCs as adult stem cells are free from ethical concerns, residents of multiple tissues, able to efficiently differentiate along an osteogenic lineage, possess non-immunogenic properties, have injury-seeking capabilities, and can be used as vehicles for bone gene therapy. These characteristics make MSCs safe and promising candidates for use in bone engineering and regeneration. Currently, several clinical trials are being performed on problematic human bone lesions, including nonunion fractures, delayed union, bone cysts, and bone neoplasms, among others. These ongoing registered trials are available at the following clinical trial website: http://clinicaltrials.gov/.
